# Colour vision deficiency in anaesthetic areas

**DOI:** 10.1016/j.bjao.2026.100536

**Published:** 2026-02-17

**Authors:** Jeremy Webb, Benjamin J. Blaise

**Affiliations:** 1 Department of Paediatric Orthopaedic Surgery, Evelina London Children’s Hospital, Guy’s and St Thomas’ NHS Foundation Trust, London, UK; 2 Department of Paediatric Anaesthesia, Evelina London Children’s Hospital, Guy’s and St Thomas’ NHS Foundation Trust, London, UK; 3 Centre for the Developing Brain, St Thomas’ Hospital, King’s College London, London, UK

**Keywords:** colour blindness, colour vision deficiency, inclusivity, paediatric anaesthesia, theatre hats

Editor

Inclusivity is a key value of our healthcare system. When asked “Is your name Dr. Andy?” by a staff member in the operating theatre complex, an anaesthetist replied: “No, I’m Dr. Ben, it’s written on my hat” with a surprised look. The colleague eventually replied: ‘Sorry, I cannot read your name on this background’ (Fig. 1a).

**Fig 1:**
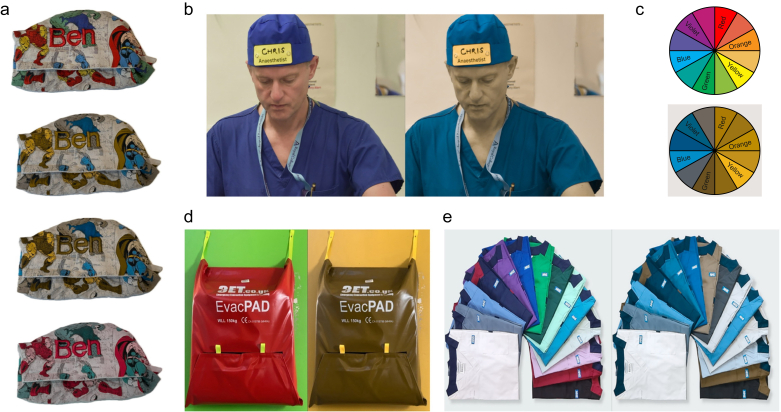
(a) Potentially problematic theatre hat for a deuteranope person, with red embroidered letters on a red and green background. top: original hat, middle top, middle bottom, and bottom: same hat visualised using a chromatic vision simulator (CVSimulator) app simulating deuteranopia, protanopia, and tritanopia, respectively. In this case, the red embroidery can merge with the background (Hulk’s green limbs, Iron man’s red armour, and Thor’s red cloak). (b) Eco Ninjas® reusable surgical hat with high-contrast name and role tag pinned on it (image reproduced with authorisation from Dr Whiten and the Communication Department at the Musgrove Park Hospital, Somerset NHS Foundation Trust). Left normal vision, right simulated deuteranopia. (c) Colour wheel viewed with normal colour vision (top) and visualised through a chromatic vision simulator for people with deuteranopia (bottom). (d) Emergency equipment available in the theatre environment at Evelina London Children’s Hospital with normal vision (left) showing clear contrast, whereas simulated deuteranopia (right) reveals an equipment merging in the background. (e) New NHS uniform colours viewed with normal vision (left) and simulated deuteranopia (right).

One in 12 males has congenital colour vision deficiency (CVD), whereas females are less affected (one in 200). It can also be acquired. CVD is a disorder of the visual system, specifically relating to colour perception, resulting in reduced or altered ability to distinguish between different colours and shades. CVD has been defined as ‘a visual impairment, characterised by the inability to perceive colours in the same way as those with normal colour vision’.^1^ However, there is limited awareness and adaptation of the clinical environment to this non-visible and under-recognised disability.

CVD is a spectrum. Standard colour vision requires three sets of cones on the retina (trichromacy). If a full set of cones is missing, this is referred as dichromacy: deuteranopia (lacking sensitivity to green light owing to absent M-cones, Fig. 1a middle top), protanopia (lacking sensitivity to red light owing to absent L-cones, Fig. 1a middle bottom), tritanopia (lacking sensitivity to blue light owing to absent S-cones Fig. 1a bottom). If cones are partially working, the suffix -anomaly replaces -opia. Achromatopsia/monochromacy corresponds to the complete loss of all three sets of cones, resulting in a black and white vision using rod photoreceptors alone.^2^

The ability to distinguish colour is also affected by the environment and whether the light is natural or artificial. We asked our colleague with CVD to look at Dr. Ben’s 17 surgical hats, only three were difficult to read. However, it is hard to predict which colour combinations will be difficult or impossible to read and this can vary from one individual to another.^1^

CVD, no matter how mild or severe, is challenging and people with this condition might not be eligible for certain careers. Restrictions vary from one country to another; in the UK, they exist mainly in the armed forces, police, fire services, electrical engineering, and transport industries.^3^ Recent efforts have been made to support people with CVD. Additional clinical tests have been introduced in the rail industry: any applicant failing the Ishihara test will be asked to undergo a more detailed colour vision assessment, giving them an extra 47% chance to become a train driver.^4^ A similar two-step approach is used for electricians. Many strategies have been advised (double indicators, high contrast kits, drawings, and documentation) to handle previously used cable colours, wiring or panel indicators. Restrictions are usually put on holding ammunitions or tasers in the armed forces.

Some South-Asian countries bar young adults with CVD from entering medical schools.^5^ There are no such restrictions in most countries. Despite recommendations, awareness is limited and mitigation strategies may be underutalized to accommodate the needs of people with CVD in healthcare.^6^^,^^7^

Our operating theatre hat example is only the tip of the iceberg. Many hospitals recommend displaying name tags using high contrast options (black on white or yellow). However, these badges are not always worn or easily visible in the operating theatre environment. Hats displaying names and roles are great options, especially in critical situations to facilitate identification and closed-loop communication. Some NHS trusts introduced black ink over yellow background pads that can be clipped on reusable surgical hats (Musgrove Park Hospital in Somerset using sustainable Eco Ninjas® hats; Fig. 1b).

In its expert guidance on *Colour and contrast for people with sight loss*, the Royal National Institute of Blind people recommends avoiding complementary colours, unless there is enough tonal contrast (brightness difference) between them (Fig. 1c top).^8^ However, a simple chromatic vision simulator shows that this advice is not satisfactory and could be misleading (Fig. 1c bottom). The only safe high-contrast combinations of colours are black/white/blue/yellow. The Web Content Accessibility Guidelines on colour contrast should always be followed.^9^

Much of our anaesthetic equipment is colour coded, such as drug stickers, i.v. or arterial cannula, oropharyngeal airways, blood collection tubes, ECG leads, and cables. It has been decided that changing drug stickers would introduce more confusion and potential risks.^10^ A patient monitor screen is harder to read for staff with CVD. Our patient trolleys/carts have an emergency red handle to place a patient in Trendelenburg, near a yellow one to lift the back rest. A staff member with CVD could struggle to identify these correctly.

Furthermore, there are more critical elements that could pose potential threat to patient safety. Emergency call systems, fire and evacuation equipment on walls painted without sufficient contrast (Fig. 1d), and guidelines where important sections are highlighted in red and green, are indistinguishable for staff with CVD. Shock delivering button on a typical St John Ambulance defibrillator is visible (red button with a lightning on a green background) but much more difficult to find for a staff with CVD. Luckily enough, there is a second indicator (the lightning), and the shock delivery does not rely only on a colour code.

Staff with CVD often minimise the impact of CVD in their life and will have developed coping strategies. There is no or really limited research on links between CVD and harmful events despite clear risk identification.^6^ An issue is the variety of guidance and the absence of a clear and unified policy providing a consistent approach across healthcare. The introduction of new equipment using a colour code that may not be accessible to staff with CVD induces a potential risk to patient safety. Colour should always be used in a universally accessible way and consistent standards available across the health service. Given the CVD sex ratio, it also constitutes an indirect sex discrimination, having a disproportionate impact on males to make errors when performing certain tasks.

Efforts are being made to raise awareness and mitigate CVD in non-clinical areas. Designers have imagined new traffic lights, which are unfortunately not often used. Grassports Australia have issued guidance to make the field lines more visible on sport surfaces. World Rugby has introduced a directive to avoid or limit kit clashes (situation where players with CVD cannot distinguish two sport jerseys). Some clashes are still happening owing to kit availability. The universal guidance on accepted colour contrast is unfortunately not followed by the NHS.

The introduction of colour-coded NHS healthcare uniforms (Fig. 1e) is likely to make it impossible for some people with CVD to differentiate between healthcare professionals and interact with the right staff (https://www.supplychain.nhs.uk/categories/facilities-and-office-solutions/uniforms/nhs-healthcare-uniform/). The nuances of white, beige, and light green, or purple and blue, become difficult or impossible to distinguish for people with CVD.

At our individual level, we should be mindful of people with CVD. Our default colour coding systems may be difficult or impossible for people with CVD to see. Recommended high-contrast colours should be used, and the result checked with a chromatic vision simulator. A holistic approach should be implemented throughout our healthcare systems to ensure everyone understands that CVD is a protected characteristic. This should include actionable and auditable steps: mandatory second indicators, procurement standards, and staff awareness training.

## Authors’ contributions

Drafting of paper: both authors

Critical review of paper: both authors

Writing of final version of paper: BJB

Approval of final version of paper: both authors

## Declaration of interests

The authors declare that they have no conflicts of interest.
